# Anti-3-Hydroxy-3-Methylglutaryl Coenzyme A Reductase (Anti-HMG CoA) Myopathy With Cardiac Involvement: Presentation, Diagnosis, and Management

**DOI:** 10.7759/cureus.23125

**Published:** 2022-03-13

**Authors:** Lisa Liu, Steven Tessier, Firas Ido, Santo Longo, Sudip Nanda

**Affiliations:** 1 Department of Pathology, St. Luke's Hospital, Bethlehem, USA; 2 Department of Pulmonary and Critical Care Medicine, St. Luke's Hospital, Bethlehem, USA; 3 Department of Cardiology, St. Luke's Hospital, Bethlehem, USA

**Keywords:** autoantibodies, immune-mediated necrotizing myopathy, statin, cardiac involvement, myopathy

## Abstract

Immune-mediated necrotizing myopathy (IMNM) is categorized into three groups: anti-3-hydroxy-3-methylglutaryl coenzyme A reductase (HMGCR) IMNM, anti-signal recognition particle (SRP) IMNM, and seronegative IMNM. Cardiac involvement has been reported in a significant segment of patients with IMNM of the anti-SRP type. Emerging evidence now suggests that cardiac involvement is also implicated in the anti-HMGCR subgroup. In this report, we present a case of anti-HMGCR IMNM with cardiac involvement demonstrated by elevated troponin levels, a low ejection fraction of 40%, and regional wall motion abnormalities in the inferior, inferolateral, anteroseptal, inferoseptal, and anterolateral myocardial walls, as visualized on echocardiography. These findings markedly improved after treatment with intravenous immunoglobulin (IVIG) and prednisone. This case and other recent reports highlight the need for a cardiac workup in patients diagnosed with anti-HMGCR IMNM.

## Introduction

Statins are commonly prescribed as lipid-lowering medications, and they function by interfering with cholesterol synthesis. Their mechanism of action involves the inhibition of the rate-limiting enzyme 3-hydroxy-3-methylglutaryl coenzyme A reductase (HMGCR). The expression of HMGCR is upregulated in muscle cells following exposure to statins. In addition, regenerating muscle cells express high levels of HMGCR necessary for normal muscle cell differentiation [1]. However, in rare cases, the use of statins can lead to an immune-mediated necrotizing myopathy (IMNM), a recently recognized subtype of idiopathic inflammatory myopathies [2-4]. While the original categorization of idiopathic inflammatory myopathies included polymyositis and dermatomyositis, a new classification system was developed by the European Neuromuscular Centre in 2003 and later expanded in 2016 to include anti-HMGCR IMNM, anti-signal recognition particle (SRP) IMNM, and seronegative IMNM [5,6].

The pathophysiology of statin-induced-myopathy is associated with the binding of anti-HMGCR antibodies to the surface of myofibers, resulting in complement activation, recruitment of macrophages, and muscle cell necrosis [7]. Furthermore, these antibodies impair muscle regeneration by disrupting myoblast differentiation [8]. Anti-HMGCR IMNM clinically presents with progressive proximal muscle weakness that correlates with elevated creatinine kinase (CK) levels (>1,000-10,0000 U/L), indicating skeletal muscle damage [7]. While the anti-HMGCR subgroup was previously thought to lack cardiac involvement [8,9], a recent retrospective study found that 11 out of 36 patients with anti-HMGCR IMNM had echocardiographic abnormalities [10]. In this report, we discuss a case of anti-HMGCR IMNM showing cardiac involvement with elevated cardiac troponins, a low ejection fraction, and extensive regional wall motion abnormalities.

## Case presentation

A 68-year-old female with a history of childhood epilepsy, hypothyroidism, hyperlipidemia, and prior falls was brought to the emergency department (ED) after falling twice. The first fall had occurred while the patient had been getting out of bed in the morning and the second while the patient had bent over to grab a newspaper. The patient denied losing consciousness or injury to the head. She also denied muscle pain, lightheadedness, or dizziness. The patient had had four ED visits in the past year due to falls. Her medications included atorvastatin 40 mg, which had been started five years prior. The physical exam was unremarkable except for proximal weakness (3/5) in the shoulders and hip flexors. The patient’s fine motor coordination skills were intact. She was able to follow commands, conversed without difficulty, made eye contact, and was oriented to person, place, time, and year. The patient did not have any tremors, seizures, facial asymmetry, or speech difficulty, and the sensation was intact. Her vital signs were as follows: blood pressure of 143/61 mmHg, a pulse rate of 92 beats/minute, temperature of 99.5 °F, respiratory rate of 18 breaths per minute, oxygen saturation of 96% on room air, and a body mass index of 29 kg/m^2^. The complete blood count and differentials were within normal limits except for elevated neutrophils at 88% (reference range: 40-60%) and decreased lymphocytes at 9% (reference range: 20-40%). A metabolic panel revealed an elevated alanine transaminase level of 123 U/L (reference range: 7-55 U/L) and an aspartate aminotransferase level of 308 U/L (reference range: 8-33 U/L). Vitamin B12 levels were less than 60 pg/mL (reference range: 100-900 pg/mL). Serum CK levels were obtained and found to be elevated at 3575 U/L (reference range: 26-192 U/L). The CK MB isoenzyme (CK-MB) index was 3.0% (reference range: 0.0-2.5%), indicating the involvement of skeletal and cardiac muscles. Troponin levels were not checked during this hospital stay. An electrocardiogram performed on the day of admission showed a normal sinus rhythm with non-specific ST and T wave abnormalities in the lateral leads. The patient was started on B12 injections of 1000 μg intramuscular daily. She was also recommended to follow up with neurology for an electromyogram and was discharged to a short-term rehabilitation facility for physical therapy.

Three weeks later, the patient returned to the ED following another fall. She reported diffuse muscle weakness that limited her ability to ambulate without assistance, get out of a car, walk up steps, and comb her hair. On physical exam, she had difficulty rising from the chair. Grip and biceps strength were relatively preserved with the exception of the left hand, which was weaker. The patient had significantly decreased bilateral triceps and deltoid strength. The CK level was 4805 U/L and CK-MB index was 3.7%. The troponin I level was normal although this was only checked once. Vitals were stable and other lab values were similar to those from the previous admission. CT of the head showed no acute abnormality and the chest X-ray was unremarkable. Atorvastatin was discontinued due to a concern for rhabdomyolysis and elevated liver enzymes. The patient received aggressive intravenous fluid hydration due to rhabdomyolysis.

**Figure 1:**
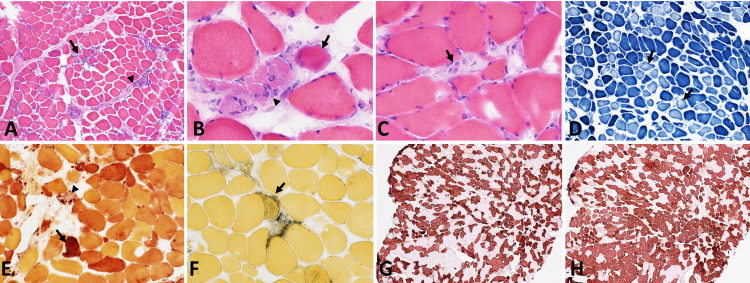
Histological stainings of the muscle biopsy (A) Hematoxylin and eosin (H&E) showed focal sparse chronic lymphoid inflammation within the perimysium (arrowhead) and within the endomysium to a lesser degree (arrow). (B) Myophagocytosis, phagocytosis of muscle fibers by large multinucleated cells (arrowhead), basophilic regenerating muscle fibers (arrow), and (C) pale, necrotic muscle fibers (arrow) are noted. (D) Nicotinamide adenine dinucleotide tetrazolium reductase (NADH-TR) stain shows paler staining cells representing necrotic muscle fibers (arrows). (E) Esterase stain dark highlights in a few of the atrophic muscle fibers (arrow) and macrophages were seen in association with necrotic muscle fibers (arrowhead). (F) Alkaline phosphatase shows hemophagocytic cells (arrow), which are macrophages or histiocytes that phagocytose erythrocytes or lymphocytes. (G, H) Immunohistochemical stain for myosin heavy chain fast and slow (MHCf and MHCs) demonstrate atrophic muscle fibers of both myofiber types

A myositis serologic panel was negative for ribonucleoprotein (RNP), Mi-2, Ku, SRP, threonyl (PL-7), alanyl (PL-12), glycyl (EJ), isoleucyl (OJ), and histidyl (Jo-1). An electromyogram of the right upper and lower extremities later showed a generalized myopathic process with membrane instability, axonal polyneuropathy, and moderate right carpal tunnel syndrome. Anti-acetylcholine receptor (AChR) antibody and anti-HMGCR antibody tests, as well as a muscle biopsy, were obtained. The patient’s AChR binding antibody, AChR blocking antibody, and AChR modulating were negative. However, the anti-HMGCR antibody was positive with a level of 94.5 CU (reference level: <20.0 CU). The muscle biopsy revealed scattered regenerating and necrotic muscle fibers with a limited inflammatory infiltrate (Figure 1).

The presence of anti-HMGCR antibody combined with muscle weakness and elevated CK levels strongly supported a diagnosis of anti-HMGCR IMNM.

Two weeks after the muscle biopsy, the patient presented again to the ED with generalized weakness after yet another fall. Physical exam this time revealed mild lower extremity edema. The CK was 3097 U/L and CK-MB index was 7.8%. Troponin I levels were elevated and peaked at 2.36 ng/mL (reference range: <0.04 ng/mL).

An echocardiogram showed a left ventricular ejection fraction of 40% in addition to mid to apical regional wall motion abnormalities in the inferior, inferolateral, anteroseptal, inferoseptal, and anterolateral myocardial walls. A prior echocardiogram performed six years ago had shown an ejection fraction of 65% with normal left ventricular chamber size, normal systolic and diastolic function, and no regional wall motion abnormalities. The patient did not want to receive a left heart catheterization. She received intravenous immunoglobulin (IVIG) of five cycles and was started on prednisone 60 mg daily for the IMNM. Ten days after admission, the CK levels decreased to 937 U/L and the CK-MB index decreased to 6.0%.

**Figure 2:**
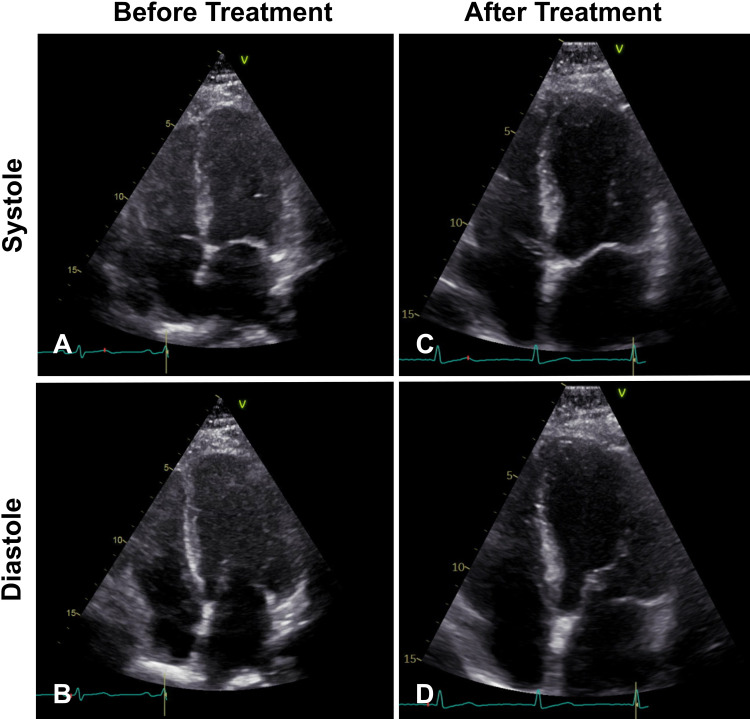
Echocardiograms before and after treatment Echocardiograms of patient’s heart before treatment (ejection fraction: 48%) (A, B) and after treatment (ejection fraction: 60%) (C, D) in systole and diastole, respectively

On follow-up two months later, the patient showed an improvement in symptoms and had not sustained any additional falls. She was started on azathioprine 25 mg twice daily and was to be slowly tapered off prednisone. A repeat echocardiogram showed the resolution of the cardiomyopathy with an ejection fraction of 60% and no regional wall motion abnormalities. Images of the patient’s echocardiogram comparing systole and diastole before the treatment with those after the treatment are illustrated in Figure 2. Videos of the patient’s echocardiogram before and after the treatment are shown in Videos 1, 2, respectively. Notably, there was a decreased movement of the heart apex before the treatment compared with after the treatment. 

**Video 1:** Echocardiogram before the treatment

**Video 2:** Echocardiogram after the treatment

The patient has remained asymptomatic and without any hospitalizations for additional falls so far. The patient’s clinical course is illustrated in Table 1.

**Table 1:** Timeline of patient's treatment course, laboratory values, and imaging results Reference values: CK: 26-192 U/L; troponin I: ≤0.04 ng/mL; INR: 0.84-1.19; EF: 50-75% ↑ represents values above the reference range, and ↓ represents values below the reference range. An empty column represents a gap in the timeline *Average of four values. **Average of three values, at a different visit Ab: antibody; AChR: acetylcholine receptor; CK: creatine kinase; disc: discontinued; Echo: echocardiogram; ED: emergency department; EF: ejection fraction; HMGCR: 3-hydroxy-3-methylglutaryl coenzyme A reductase; IVIG: intravenous immunoglobulin

	March 2014	September 2014	April 2015		January 2020	February 2020	March 2020	April 2020	May 2020	June 2020	July 2020	August 2020	September 2020	October 2020	November 2020
Statin use	Started on simvastatin		Switched to atorvastatin							Statin disc					
CK (U/L)									↑3575	↑4805 ↑3062 ↑2856		↑3,097 ↑937		↑932	
INR										1.06		↑1.24			
Troponin I (ng/mL)										<0.02		↑1.92* ↑1.55**			
Echo: EF		65%										↓40% ↓48%		60%	
ED visit due to fall						X	X	X	X	X		X			
EMG											X				
HMGCR, AChR Ab test											X				
Muscle biopsy												X			
Initiated on IVIG												X			
Initiated on prednisone												X			
Initiated on azathioprine															X

## Discussion

Multiple factors suggest that this patient’s cardiac involvement was secondary to IMNM. Firstly, the patient’s transient low ejection fraction of 40% and regional wall abnormalities seen on echocardiogram had not been noted six years prior, and these had preceded the detection of anti-HMGCR antibodies. In addition, the patient’s cardiac dysfunction coincided with the presence of muscle weakness and disease progression. Finally, the treatment with IVIG and prednisone improved the symptoms and laboratory tests (i.e., CK). It also led to the resolution of the cardiac abnormalities and restoration of the ejection fraction. The degree of pathological cardiac involvement could not be further assessed since a cardiac biopsy was not performed. Furthermore, there was no cardiac MRI to assess for scarring. However, given the normal follow-up echocardiogram, it is unlikely that the patient developed permanent damage.

Anti-HMGCR IMNM typically presents with proximal muscle weakness and elevated CK levels [7]. Cardiac involvement in IMNM is not typical and has been limited to small case series so far in the literature [8,9]. Recently, a retrospective study investigating 109 patients with IMNM, who were evaluated for echocardiogram abnormalities, found that of the 36 patients positive for anti-HMGCR antibody, 11 showed left ventricular diastolic dysfunction, and six showed systolic dysfunction [10]. Another study documented cardiac abnormalities in patients with IMNM, although the study did not specify the IMNM subgroups [11]. Our patient showed regional wall motion abnormalities in the inferior, inferolateral, anteroseptal, inferoseptal, and anterolateral myocardial walls on echocardiography, which were reversible following the treatment of IMNM.

A diagnosis of anti-HMGCR IMNM is confirmed when the following three criteria are met: (1) elevated serum CK levels, (2) proximal muscle weakness, and (3) anti-HMGCR antibodies [6]. Initial treatment includes intravenous and/or oral steroids along with the addition of IVIG and/or methotrexate concurrently or within one month [6]. After at least two years of well-controlled disease with minimal or no steroids, other agents should be stopped or tapered off, although many patients do require prolonged IVIG.

This case of cardiac involvement in the anti-HMGCR subgroup and the emerging evidence reviewed herein suggest that a cardiac evaluation should be considered in patients diagnosed with anti-HMGCR IMNM.

## Conclusions

Cardiac involvement is a rare finding in anti-HMGCR IMNM. We reported the case of a patient with anti-HMGCR IMNM with cardiac involvement demonstrated by elevated troponin levels, a low ejection fraction, and regional wall motion abnormalities in the inferior, inferolateral, anteroseptal, inferoseptal, and anterolateral walls on echocardiography. The patient's condition markedly improved after treatment with IVIG and prednisone. This case underlines the need for a cardiac workup in patients diagnosed with anti-HMGCR IMNM.
